# Kinesthetic vs. visual focus: No evidence for effects of practice modality in representation types after action imagery practice and action execution practice

**DOI:** 10.1016/j.humov.2023.103154

**Published:** 2023-10-14

**Authors:** Stephan F. Dahm, Martina Rieger

**Affiliations:** a Universität Innsbruck, Department of Psychology, Innsbruck, Austria; b UMIT TIROL – Private University of Health Sciences and Health Technology, Institute of Psychology, Hall in Tyrol, Austria

**Keywords:** Motor imagery, Action imagery practice, Visual-spatial representations, Effector-dependence, Serial reaction time task

## Abstract

Action-imagery practice (AIP) is assumed to result in partly different action representations than action-execution practice (AEP). The present study investigated whether focusing on either kinesthetic or visual aspects of a task during practice amplifies or diminishes such differences between AIP and AEP. In ten sessions, four groups, using either AIP or AEP with either kinesthetic or visual focus, practiced a twelve-element sequence in a unimanual serial reaction time task. Tests involved the practice sequence, a mirror sequence, and a different sequence, each performed with the practice and transfer hand. In AIP and AEP, in both hands, reaction times (RTs) were shorter in the practice sequence than in the different sequence, indicating effector-independent visual-spatial sequence representations. Further, RTs were shorter in the practice hand than in the transfer hand in the practice sequence (but not in the different sequence), indicating effector-dependent representations in AEP and AIP. Although the representation types did not differ, learning effects were stronger in AEP than in AIP. Thus, although to a lower extent than in AEP, effector-dependent representations can be acquired using AIP. Contrary to the expectations, the focus manipulation did not have an impact on the acquired representation types. Hence, modality instructions in AIP may not have such a strong impact as commonly assumed, at least in implicit sequence learning.

## 1 Introduction

Action-imagery refers to the mental simulation of an action without performing actual movements (Krüger, Hegele, & Rieger, 2022; Rieger, Boe, Ingram, Bart, & Dahm, 2023). Action-imagery practice (AIP, also called ‘mental practice’ or motor imagery practice’) refers to the repetitive use of action-imagery with the aim to improve subsequent action-execution. Indeed, AIP has been shown to improve subsequent action-execution. However, performance improvements are lower than after action-execution practice (AEP, also called ‘physical practice’) (Ladda, Lebon, & Lotze, 2021; Lindsay, Larkin, Kittel, & Spittle, 2023; Simonsmeier, Androniea, Buecker, & Frank, 2021; Toth, McNeill, Hayes, Moran, & Campbell, 2020). However, the mechanisms behind the acquisition of action representations that lead to performance improvements and which types of actions representations are acquired in AIP have not been fully uncovered (Dahm et al., 2023; Frank, Kraeutner, Rieger, & Boe, 2023; Kraeutner, Cui, Boyd, & Boe, 2022; Rieger et al., 2023). Further, what is learned in a certain task, partly seems to depend on which modality is encouraged to utilize in the imagery instructions (Callow, Jiang, Roberts, & Edwards, 2017; Féry, 2003; Krüger et al., 2022). The most common modalities in action imagery are kinesthetic imagery (how it feels like performing the action) and visual imagery (how it looks like performing the action) (Dahm, 2020). Therefore, in the present study, we investigated whether the type of practice (AIP vs. AEP) and modality focus during practice (kinesthetic vs. visual) result in the acquisition of different representations.

Action imagery is assumed to involve similar mechanisms to optimize and predict action consequences as they are used in action execution (Dahm, Weigelt, & Rieger, 2023; Grush, 2004; Jeannerod, 2001; Rieger et al., 2023). This has been supported by brain imaging studies showing similar activation in motor-related brain areas during imagery and execution (Lorey et al., 2013; Sobierajewicz, Przekoracka-Krawczyk, Jaśkowski, Verwey, & van der Lubbe, 2017). Further support comes from behavioral studies, showing that imagination durations and execution durations are influenced similarly by both cognitive and motor constraints (Dahm & Rieger, 2016a; Dahm & Rieger, 2016b; Guillot & Collet, 2005).

It is assumed that, to achieve intended effects, inverse models select motor commands when imagining or executing an action (Rieger et al., 2023). The motor commands contain information about the quantity and timing of muscle-groups. In both, action execution and action imagery, an efference-copy (Rieger et al., 2023) is built, which is used by forward models to predict the action outcomes (Wolpert, Diedrichsen, & Flanagan, 2011; Wolpert, Ghahramani, & Flanagan, 2001). Possibly, this contributes to the dynamic perceptual effects (e.g., images, feelings) during action imagery. Of course, action imagery differs from action execution in such a way that actual action outcomes do not occur in action imagery. For this, it is necessary to inhibit the activation of the effectors, for instance by blocking the motor commands to reach the muscle-groups (Guillot, Di Rienzo, Macintyre, Moran, & Collet, 2012; Rieger, Dahm, & Koch, 2017). While action imagery and action execution are assumed to be similar during the planning phase of actions, the underlying mechanisms may differ during the execution phase of actions (Glover & Baran, 2017). While ‘planning errors’ occur equally often, ‘movement errors’ occur less often in action imagery than in action execution (Dahm & Rieger, 2019; Rieger, Martinez, & Wenke, 2011), which might be due to the lack of actual feedback after action imagery. But still, performance enhancements in AIP are not only due to visual-spatial learning and movement planning, but extend to performance enhancements based on motor mechanisms in the movement execution phase (Dahm & Rieger, 2023; Ingram, Solomon, Westwood, & Boe, 2019), although performance enhancements are lower than in AEP (Dahm, Weigelt, & Rieger, 2023).

This goes along with the observation that partly different types of representations are acquired in AIP and AEP. For instance, more effector-dependent representations and effector-independent intrinsic representations are acquired in AEP than in AIP while effector-independent visual-spatial representations are equally acquired in AEP and AIP (Amemiya, Ishizu, Ayabe, & Kojima, 2010; Dahm, Weigelt, & Rieger, 2023; Kraeutner, McArthur, Kraeutner, Westwood, & Boe, 2020; Wohldmann, Healy, & Bourne Jr., 2008). *Effector-dependent* representations involve motor commands that are solely available to the practiced effectors (Imamizu & Shimojo, 1995; Panzer, Krueger, Muehlbauer, Kovacs, & Shea, 2009), observable in better performance in the practice hand than in the transfer hand in intermanual transfer paradigms (Dahm, Weigelt, & Rieger, 2023). *Effector-independent intrinsic* representations involve motor commands that are body-based and therefore available to the homologous effectors (Criscimagna-Hemminger, Donchin, Gazzaniga, & Shadmehr, 2003), observable in better performance in mirror actions in the transfer hand than in unpracticed control actions in the transfer hand (e.g., different or random sequences). *Effector-independent visual-spatial* representations involve motor commands that are environment-based, but not restricted to the effectors (Imamizu & Shimojo, 1995), which benefit all actions (in the practice and transfer hand) that involve the same stimulus pattern (Remillard, 2003; Soetens, Melis, & Notebaert, 2004; Verwey & Clegg, 2005) or the same response locations (Willingham, Wells, Farrell, & Stemwedel, 2000).

During action-imagery, it is possible to focus on different types of modalities (Cumming & Eaves, 2018; Dahm, 2020; Krüger et al., 2022). When imagining oneself rowing in a boat on the sea, one may perceive the rough wind blowing into one’s face (tactile imagery), one may smell the sun blocker on the skin (olfactory imagery), one may taste the saltwater of the sea on one’s lips (gustatory imagery), one may feel the muscles moving in one’s legs, body, and arms (kinesthetic imagery), and one may see the boat moving through the sea (visual imagery). In studies on AIP, particularly kinesthetic and visual imagery are investigated (Guilbert, Fernandez, Molina, Morin, & Alamargot, 2021; Yang, Jeon, Kim, & Chung, 2021) as they are the most important modalities for most actions.

Several differences between kinesthetic and visual imagery have been found (Callow et al., 2017; Féry, 2003; Guilbert et al., 2021; Lee et al., 2019; Mizuguchi, Nakamura, & Kanosue, 2017; Yang et al., 2021). For instance, vividness ratings were lower in kinesthetic imagery than in visual imagery of handwritings when performed in an incongruent hand posture (Guilbert et al., 2021). It has been observed that kinesthetic action-imagery and visual action-imagery involve partially different neural networks (Lee et al., 2019), with the networks in kinesthetic action-imagery overlapping with the networks in action-execution, and the networks in visual action-imagery overlapping with the networks in action observation (Yang et al., 2021). In line with this, corticospinal excitability increases in the primary visual cortex during visual action imagery, but not during kinesthetic action imagery (Mizuguchi et al., 2017). The combination of kinesthetic and visual AIP results in shorter slalom times than visual AIP alone (Callow et al., 2017). Visual-spatial accuracy of hand coordination improves faster and stronger in visual AIP than in kinesthetic AIP, which is reversed for temporal accuracy of hand motions (Féry, 2003; Féry & Morizot, 2000). These results indicate that participants may partly learn different aspects of a task, when performing kinesthetic and visual AIP. Thus, depending on the modality in practice, the task may be represented in different ways. The specific nature of the acquired representations when emphasizing kinesthetic or visual action elements remains unclear. Additionally, it is still uncertain how these types of representations evolve during the learning process when concentrating on a single modality in AIP.

In the present study, we investigated whether the type of practice (AIP vs. AEP) and modality focus during practice (kinesthetic vs. visual) result in the acquisition of different representations. We used an intermanual transfer paradigm, which enables the investigation of the acquisition of several different types of representations: effector-dependent representations (practice hand vs. transfer hand), effector-independent intrinsic representations (internal reference frame), and effector-independent visual-spatial representations (external reference frame).

We used a serial reaction time task, in which participants were not informed about the underlying sequential structure. Because effector-independent intrinsic representations are not always observed in sequence learning (Bird & Heyes, 2005; Dahm & Rieger, 2023; Dahm, Weigelt, & Rieger, 2023; Verwey & Clegg, 2005), two changes were made in comparison to a previous study (Dahm & Rieger, 2023). First, the stimuli were arranged in a diagonal manner (from top-left to bottom-right in one hand and from top-right to bottom-left in the other hand). By this, the stimuli in the practice and transfer hand differed. Hence, anticipatory stimulus learning for the transfer hand was prevented and learning of the response locations was expected (Koch & Hoffmann, 2000). Second, we increased the number of practice sessions (from four to ten sessions) and thereby the amount of practice (from 240 to 600 sequence repetitions), because effector-independent intrinsic representations are assumed to develop after extensive practice (Dahm & Rieger, 2023; Panzer et al., 2009). Therefore, we expected effector-independent intrinsic representations and effector-dependent representations to develop in the course of learning and to occur particularly at later stages of learning.

Because focusing on a certain modality during practice may influence the types of representations that are acquired, we instructed participants in two different ways. They were asked to either focus on kinesthetic or on visual elements during the task. We expected that a stronger focus on visual action elements during practice results in effector-independent visual-spatial representations. In contrast, focusing on kinesthetic action elements during practice may result in effector-dependent representations or effector-independent intrinsic representations. Whereas some previous studies investigated kinesthetic vs. visual focus in AIP, different focus instructions have usually not been investigated in AEP comparison groups. Therefore, we had no expectations for the AEP groups focusing on kinesthetic or visual elements, but expected differences between modality conditions particularly in the AIP groups (cf. Féry, 2003; Féry & Morizot, 2000).

## 2 Methods

### 2.1 Participants

Participants were recruited via student mailing lists and from acquaintances of students involved in data collection. Inclusion criteria were that participants were right-handed, between 18 and 35 years old, and that they had at least a moderate ability to imagine actions clearly and vividly, assessed with the computer-based German Version (Dahm, 2022; Dahm, Bart, Pithan, & Rieger, 2019) of the Vividness of Movement Imagery Questionnaire (Roberts, Callow, Hardy, Markland, & Bringer, 2008). Originally 126 participants were tested. A rigid check on data validity was performed, because participants performed the experiment in the absence of an experimenter at home at their personal laptops (Dahm, Ort, et al., 2023). For a detailed description of exclusion criteria see supplemental material. Of the analyzed 102 participants, the distribution of sex and the means and standard deviations of age, the laterality index (Oldfield, 1971), and the scores of external visual imagery, internal visual imagery, and kinesthetic imagery (assessed with the Vividness of Movement Imagery Questionnaire: Dahm et al., 2019; Roberts et al., 2008) are shown in Table 1, separately for each experimental group. All participants gave informed consent, and the study was approved by the local ethics committee.

The required sample size for the interaction between four groups and six test sessions was estimated with G*Power (Faul, Erdfelder, Lang, & Buchner, 2007). We assumed an effect size of *f* = 0.25 and correlations among repeated measures of *r* = 0.4. Alpha was set at 0.05 and the power (1-beta) at 0.8 which resulted in a minimum sample size of *N* = 92 (*n* = 23 per group).

## 3 Material and procedure

The experiment was run on participants’ personal laptops using Open Sesame Version 3.3.5 (Dahm, Ort, et al., 2023; Mathôt, Schreij, & Theeuwes, 2012). The experiment file is available at https://osf.io/puzr3/?view_only=3f7ae39519734ef8b4978f3eb7e39ecb. Participants practiced on ten consecutive days. Due to the exponential decline in reaction time learning curves (Heathcote, Brown, & Mewhort, 2000; Newell & Rosenbloom, 1981), we decided to successively increase the number of practice sessions between tests. Therefore, Session 1, Session 2, Session 4, Session 7, and Session 11 started with a test. A follow-up test was performed on average 31.5 days (*SD* = 3.7 days) after Session 11. An overview of tests and practice per session is shown in Table 2.

**Fig. 1:**
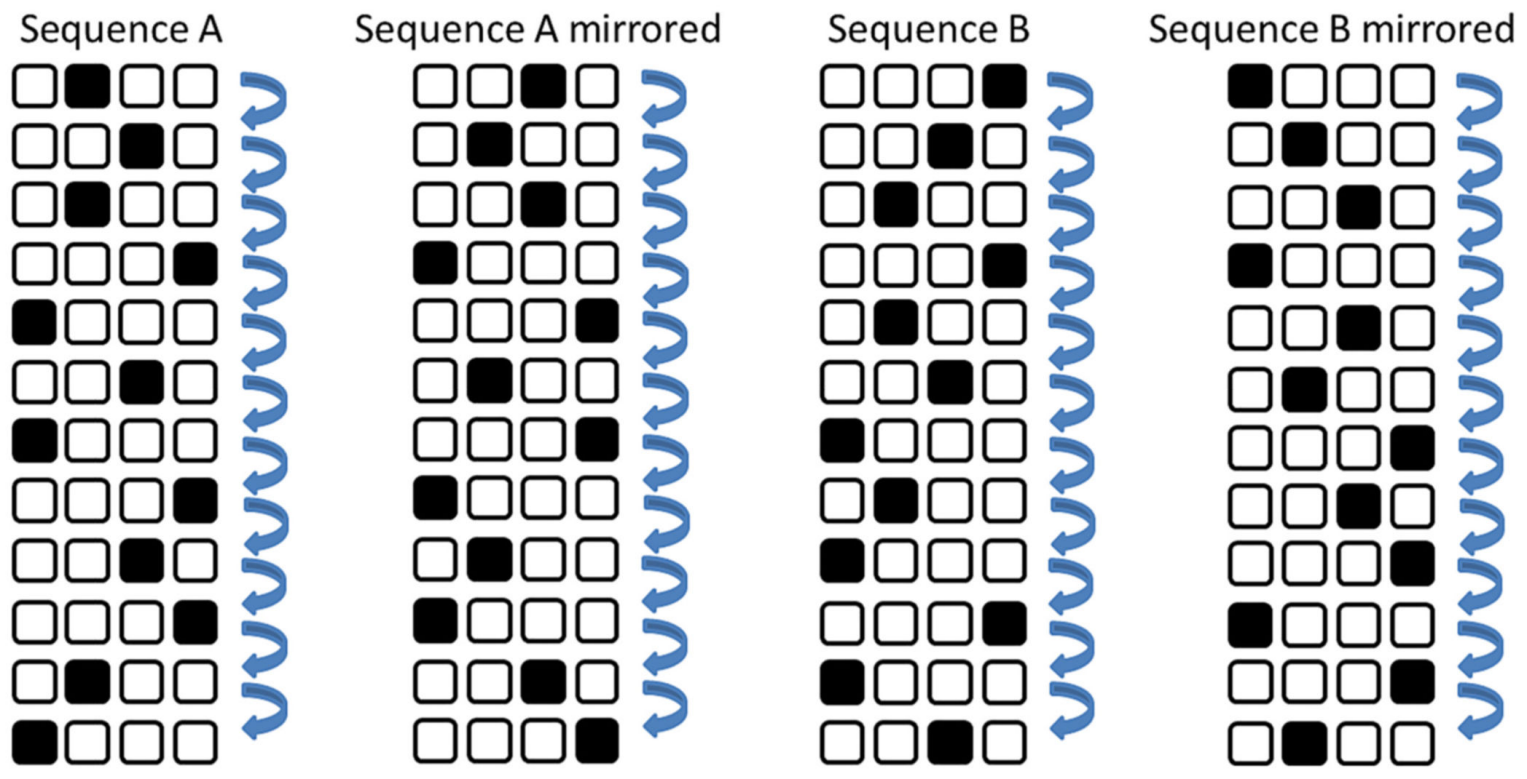
Visual illustration of the 12 responses in each sequence. All four sequences were performed during tests, but only one was performed during practice (counterbalanced across participants).

Participants performed a four-choice serial reaction time task using the index, middle, ring, and small finger of the same hand (adapted from Reber & Squire, 1998). Participants placed their index, middle, ring and little finger on the ‘F’, ‘G’, ‘H’, and ‘J’ keys. Visual stimuli consisted of four circles (*r* = 1 cm) that were arranged diagonally left top to right bottom for the left hand and left bottom to right top for the right hand (Fig. 1). An asterisk in one of the circles indicated the corresponding target button. The mapping of stimuli and responses was visual-spatially congruent with the left-most asterisk corresponding to the ‘F’ key and the right-most asterisk corresponding to the ‘J’ key.

Each sequence consisted of 12 stimuli/responses (see Fig. 1): Sequence A (GHGJFHFJHJGF), its mirrored copy (HGHFJGJFGFHJ), Sequence B (JHGJGHFGFJFH), and its mirrored copy (FGHFHGJHJFJG). In all sequences, each stimulus appeared equally often, the same stimulus did not appear on successive trials, and each stimulus transition occurred equally often. Thus, first order learning was not possible (Reber & Squire, 1998). On each series longer than five responses, the four sequences differ from each other (Bird & Heyes, 2005).

**Fig. 2:**
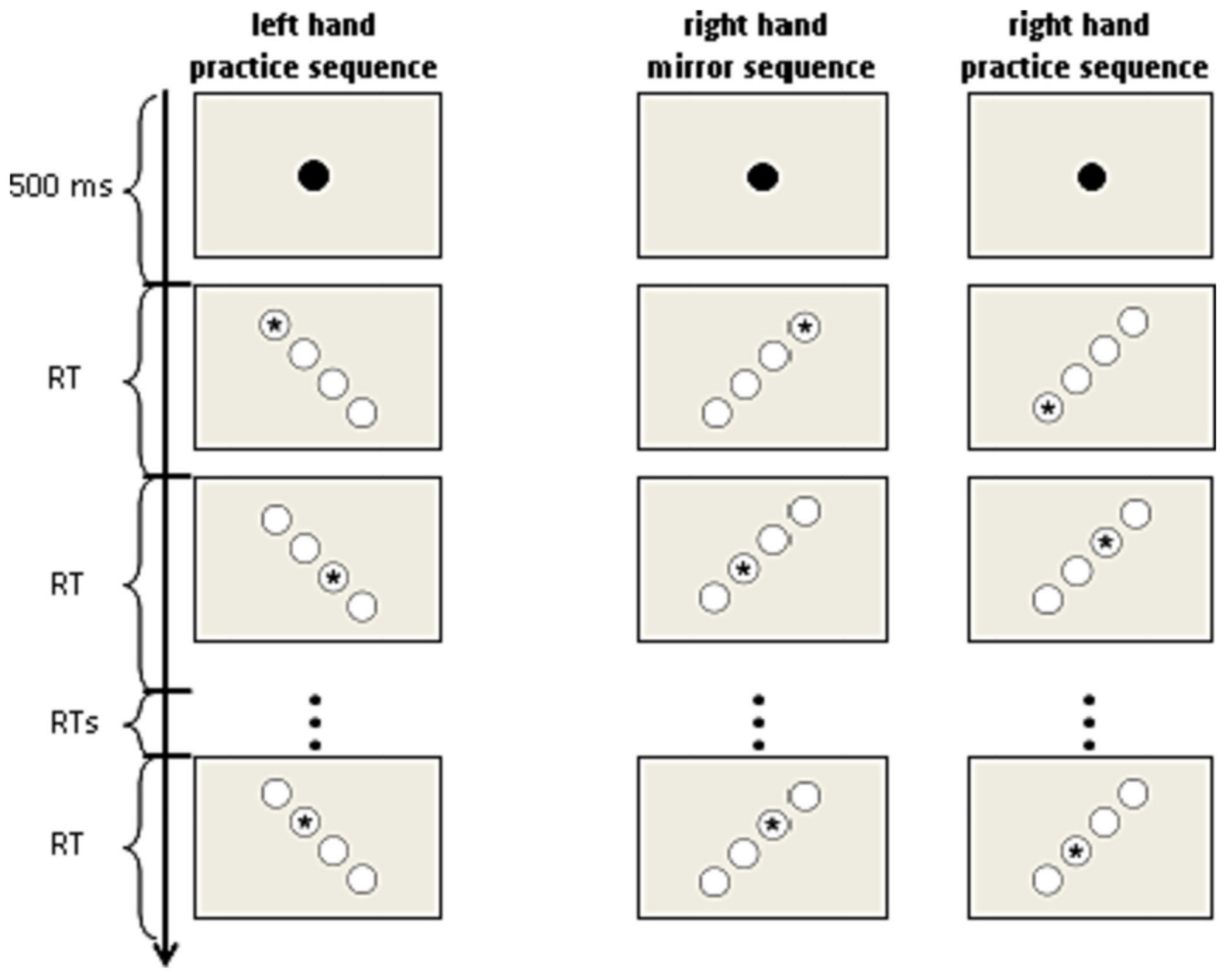
Depiction of the stimuli. A sequence started with a fixation dot. After 500 ms an asterisk appeared in one of the four circles. Immediately after a response, the asterisk appeared in another circle. Reaction time (RT) was recorded for each response.

A block of sequences started with a fixation dot. After 500 ms the first stimulus of the sequence appeared. Within a sequence the starting stimulus was random. The task was self-paced and both, correct and incorrect responses triggered the end of the stimulus. Participants responded to each asterisk by pressing the corresponding target key as fast as possible. Immediately after a response the next stimulus was presented (Fig. 2). Participants were not informed that the stimuli/responses followed a particular twelve trial sequence.

In the *tests*, the four experimental sequences were performed with each hand (left and right) resulting in eight test blocks, each involving 48 consecutive responses (i.e., sequences were repeated four times in each block). The order of the hands was blocked and counterbalanced across participants. The order of the sequences was randomized, but equal for each hand.

During *practice* participants were randomly assigned into four groups. In *AIP*, participants imagined the corresponding key press. At the very moment they imagined pressing the corresponding key, they actually pressed the shift key with the thumb of the other hand. In *AEP*, participants pressed the corresponding key and simultaneously pressed the shift key with the thumb of the other hand. In both types of practice, participants were instructed to either focus on kinesthetic or on visual aspects of the task. With *kinesthetic focus* they were instructed to focus on the feeling of the (imagined or executed) key press of the target finger. With *visual focus* they were instructed to focus on seeing the (imagined or executed) key press of the target finger through their own eyes.^1^

In all groups, practice was performed with the left hand. During practice participants performed the same sequence in all sessions (one of the four sequences counterbalanced across participants). Each practice session consisted of six blocks, each involving 120 responses (i.e., 10 repetitions of the sequence). After each block, participants received feedback about their median response times (RTs).

To check the focus manipulation, we assessed modality focus using questions in which participants rated their *focus on kinesthetic, visual, and rhythmic elements of the task*. Although not instructed, rhythmic representations were added as a neutral rating for comparison. For this, participants reported via mouse-clicks how strongly they imagined the feeling of the keypresses, seeing the key-presses, and the rhythm of the keypresses (on a rating scale from 1 – “not at all” to 9 – “very strong”) after the last practice phase in Session 10.

### 3.1 Data analysis

Dependent variables were analyzed using mixed model ANOVAs. If Mauchly’s test indicated that the assumption of sphericity was violated, we report Huynh-Feldt corrected degrees of freedom and *p*-values. Further comparisons were conducted using *t*-tests or ANOVAs with Holm adjusted pairwise comparisons (Kassambara, 2021). Statistical significance was set at *p <* .05.

As a manipulation check, we analyzed the reported kinesthetic, visual, and rhythmic focus during practice. The primary outcome variable of our experimental manipulations was the median reaction time (RT) during test blocks. RTs of the first twelve responses were excluded from each test block. Additionally, RTs of an erroneous response and its subsequent response were excluded. Analyses of further dependent variables are provided in the supplemental material, i.e., error rates, RTs during practice, and sequence knowledge. Raw data as well as the syntax for data preparation and data analyses are available at https://osf.io/puzr3/?view_only=3f7ae39519734ef8b4978f3eb7e39ecb.

## 4 Results

### 4.1 Manipulation check: modality focus questions

**Fig. 3:**
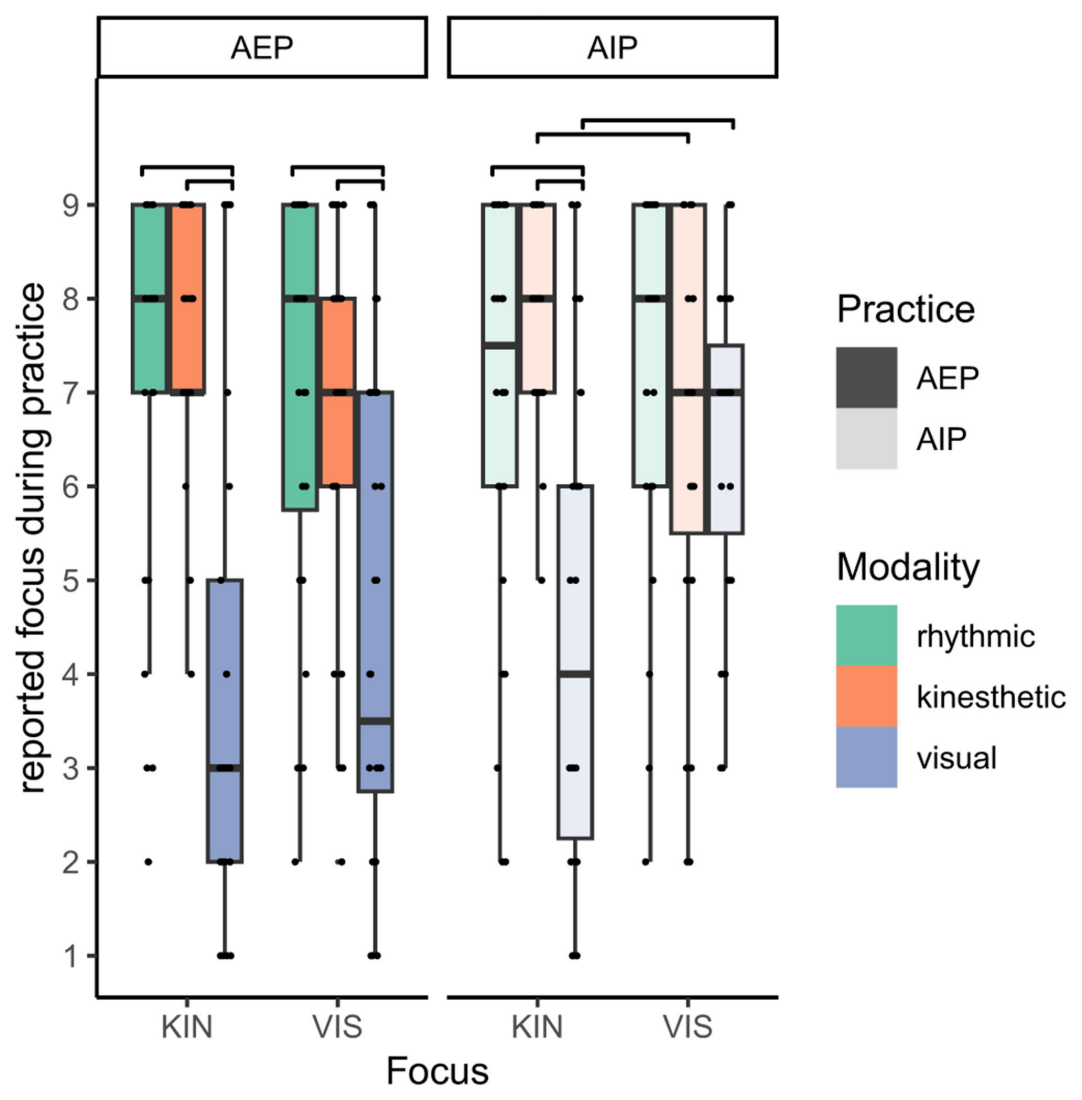
Boxplots of the reported rhythmic, kinesthetic, and visual focus during practice separately for the groups which differed in practice (AEP: action-execution practice in dark filling; AIP: action-imagery practice in light filling) and focus (KIN: kinesthetic, VIS: visual).

Boxplots of the focus ratings are shown in Fig. 3. To check whether the focus manipulation resulted in different focus ratings, a mixed-model ANOVA was calculated with the between factors practice (AIP, AEP) and focus (kinesthetic, visual) and the within factor modality (rhythmic, kinesthetic, visual). The significant main effect of modality, *F* (1.7, 163.4) = 46.1, *p <* .001, ${\text{η}}_{\text{p}}^{2}=0.32$, was modified by the interaction between modality and practice, *F* (1.7, 163.4) = 3.7, *p* = .033, ${\text{η}}_{\text{p}}^{2}=0.04$, and the interaction between modality and focus, *F* (1.7, 163.4) = 7.9, *p* = .001, ${\text{η}}_{\text{p}}^{2}=0.07$. All remaining effects were not significant: practice: *F* (1, 98) = 3.1, *p* = .081, ${\text{η}}_{\text{p}}^{2}=0.03$, practice x focus x modality: *F* (1.7, 163.4) = 1.3, *p* = .282, ${\text{η}}_{\text{p}}^{2}=0.01$, all others *F <* 1.

The visual focus rating was significantly lower than the rhythmic and kinesthetic focus ratings in all groups (*p <* .009, *d >* 0.6), except for the AIP visual focus group (*p >* .699, *d <* 0.3). The visual focus rating was significantly higher in the in the AIP visual focus group than in the AEP visual focus group (*p* = .001, *d* = 0.95), whereas the kinesthetic focus rating did not significantly differ between the AIP kinesthetic focus group and the AEP kinesthetic focus group (*p* = .300, *d* = 0.3).

Further, in AIP, the visual focus rating was stronger in the visual focus group than in the kinesthetic focus group (*p* = .002, *d* = 0.9), but not in AEP (*p* = .425, *d* = 0.2). Analogously, the kinesthetic focus rating was marginally stronger in the kinesthetic focus group than in the visual focus group in AIP (*p* = .058, *d* = 0.6), but not in AEP (*p* = .143, *d* = 0.4).

### 4.2 Reaction times in tests

**Fig. 4:**
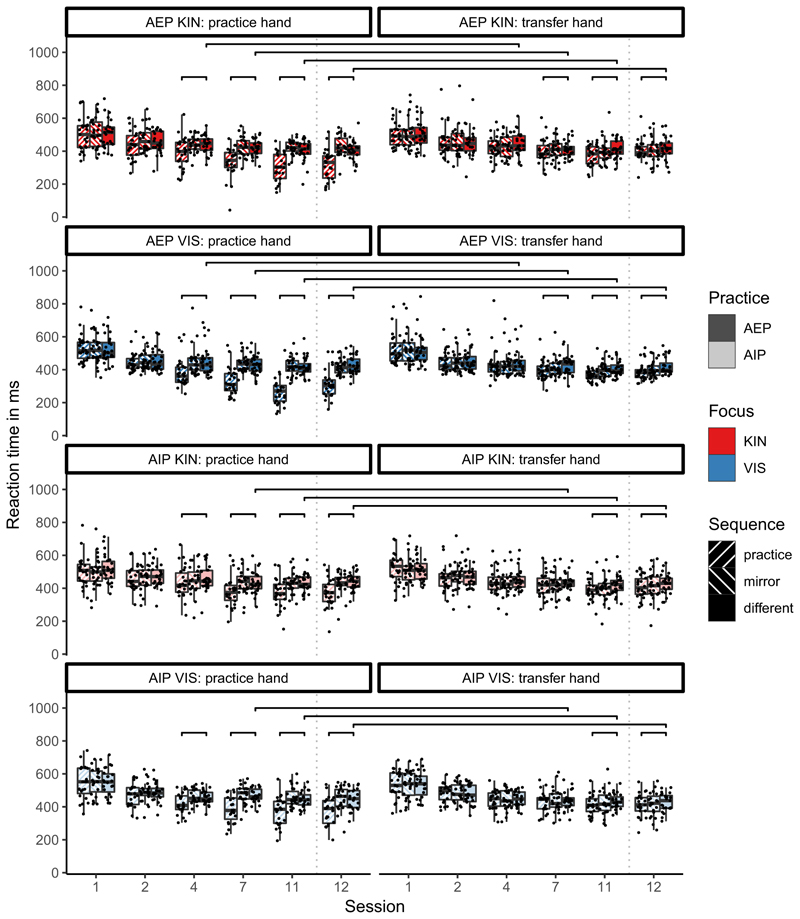
Boxplots of reaction times (in ms) depending on hand (practice, transfer), sequence (practice, mirror, different), and test (1, 2, 3, 4, 5, 6) separately for the groups which differed in practice (action-execution in dark, action-imagery in light) and focus (kinesthetic in red, visual in blue). Brackets indicate significant differences between the practice sequence and the different sequence and significant differences between the practice hand and the transfer hand in the practice sequence. (For interpretation of the references to colour in this figure legend, the reader is referred to the web version of this article.)

Boxplots of RTs are shown in Fig. 4. A mixed-model ANOVA with the between factors practice (AIP, AEP) and focus (kinesthetic, visual) and the within factors hand (practice, transfer), sequence (practice, mirror, different), and test (Sessions: 1, 2, 4, 7, 11,12) was conducted on RTs. Results of the ANOVA are shown in Table 3.

#### 4.2.1 Sequence-unspecific general learning effects and control comparisons

The significant main effect *test* indicated that RTs became significantly shorter over tests in all groups and conditions. Specifically, RTs became significantly shorter between successive tests (*p <* .001, *d >* 0.34), but became significantly longer between Session 11 and the follow-up Test in Session 12 (*p* = .001, *d* = −0.16). This effect provides evidence for sequence-unspecific learning as it occurred in all sequences.

Neither the main effect of focus nor any interactions with it became significant. The significant main effect *sequence* and the significant main effect *hand* were modified by the significant four-factor-interaction between practice, hand, sequence, and test.

##### Comparisons in Session 1 to control for group difference prior to practice

In AEP and AIP, RTs did not significantly differ between sequences (*p >* .552, *d <* 0.2). Further in AEP and AIP, RTs did not significantly differ between hands in any of the sequences (*p* = .199, *d <* 0.2). Further, RTs did not significantly differ between the practice groups in all sequences (*p* = .327, *d <* 0.2). Hence, performance did neither differ significantly between sequences, hands, nor between practice groups before practice started.

##### Comparisons of the different sequence between AEP and AIP to control for sequence-unspecific group differences in the course of learning

From Session 7 onwards RTs in the different sequence were shorter in AEP than in AIP in the (left) practice hand (*p <* .028, *d >* 0.44), but not in the (right) transfer hand (*p >* .071, *d <* 0.36). Hence, effector-dependent sequence-unspecific learning was stronger in AEP than in AIP.

#### 4.2.2 Sequence-specific learning effects

**Fig. 5:**
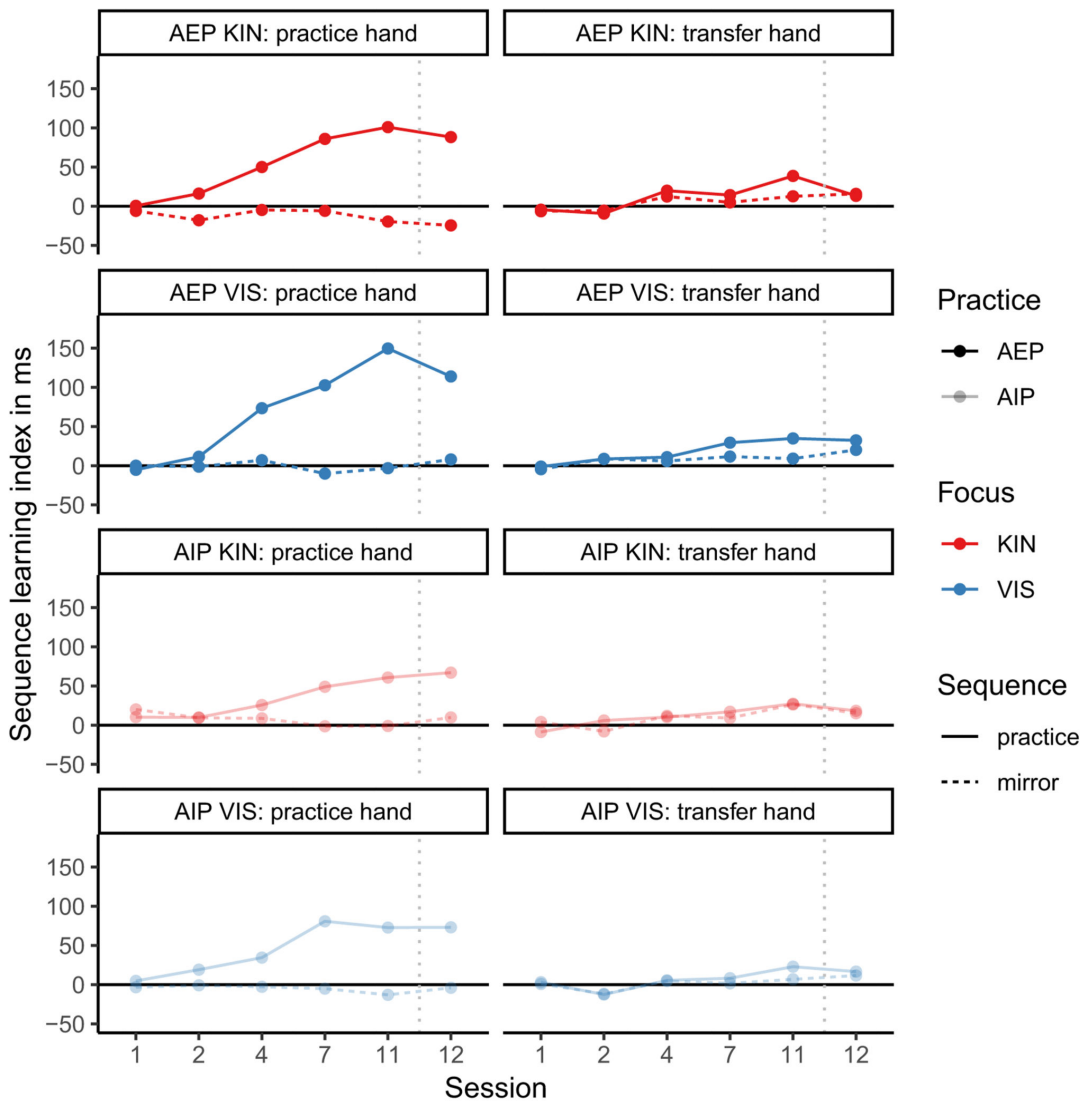
Means of the sequence learning indexes in ms (different sequence- sequence of interest) depending on hand (practice, transfer), sequence (practice, mirror), and test (1, 2, 3, 4, 5, 6) separately for the groups which differed in practice (action-execution in dark, action-imagery in light) and focus (kinesthetic in red, visual in blue). (For interpretation of the references to colour in this figure legend, the reader is referred to the web version of this article.)

To visualize sequence-specific learning effects, we calculated the sequence-learning index by subtracting the RTs of the sequence of interest (practice or mirror) from the RTs of the different sequence (Dahm & Rieger, 2023; Kraeutner, MacKenzie, Westwood, & Boe, 2016). The sequence-learning index depending on practice group, focus, and session is shown in Fig. 5.

##### After practice (in the first 10 sessions), we found the following in Session 11

In the practice hand, significantly shorter RTs in the practice sequence than in the different sequence in AEP (*p <* .001, *d* = 1.7) and AIP (*p <* .001, *d* = 0.9) indicated sequence-specific learning. Similarly in the transfer hand, RTs were significantly shorter in the practice sequence than in the different sequence in AEP (*p <* .001, *d* = 0.9) and AIP (*p <* .001, *d* = 0.9). This indicates effector-independent visual-spatial representations of the sequence.

Further, comparisons of the practice sequence between hands resulted in significantly shorter RTs in the practice hand than in the transfer hand in AEP (*p <* .001, *d* = 1.4) and AIP (*p <* .001, *d* = 0.5). In the different sequence, comparisons between hands were not significant (in AIP a tendency for the reverse effect was observed; AEP: *p* = .712, *d* = −0.05; AIP: *p* = .062, *d* = −0.3). Hence, effector-dependent representations were acquired in both practice groups. Further, in the practice sequence, the difference between practice hand and transfer hand was significantly larger in AEP (ΔRT = 88 ms) than in AIP (ΔRT = 31 ms, *p <* 001, *d* = 0.9). This shows that the effector-dependent representations were stronger in AEP than in AIP.

Comparisons of the mirror sequence and the different sequence showed that RTs in the transfer hand did not significantly differ between the sequences in AEP (*p* = .142, *d* = 0.2) and AIP (*p* = .099, *d* = 0.3) indicating no intrinsic effector-independent representations shortly after practice.

##### One-month follow-up test in Session 12

Most of the significant effects observed after practice in Session 11 were retained. However, comparisons of the practice sequence between groups in Session 12 showed that RTs in the practice sequence were significantly shorter in AEP than in AIP in the practice hand (*p* < .001, *d* = 0.7), but not in the transfer hand (*p* = .264, *d* = 0.2). This indicates stronger effector-dependent representations in AEP than in AIP after a follow-up retention interval. Additionally, in the transfer hand RTs were significantly shorter in the mirror sequence than in the different sequence in Session 12 in AEP (*p* = .003, *d* = 0.46) and in AIP (*p* = .044, *d* = 0.34). This difference in the transfer hand between the mirror and different sequence (see Fig. 5) did not significantly differ between AEP and AIP (*p* = .562, *d* = 0.12). Further, comparisons between groups in the transfer hand showed that RTs in the mirror sequence did not significantly differ between AEP and AIP (*p* = .366, *d* = 0.18). These results indicate intrinsic effector-independent representations after a break of one month in both practice groups.

##### Time course of acquisition

To investigate the time course of acquisition, we evaluated whether the effects observed in Session 11 appeared already in earlier sessions. Comparisons of the practice sequence and different sequence (sequence learning index) revealed significant differences from Session 4 onwards in AEP and AIP in the practice hand (*p <* .001, *d >* 0.7), but only from Session 7 in the transfer hand (*p >* .111, *d <* 0.3). Additionally, in the practice hand the sequence learning index in the practice sequence was significantly larger in AEP than in AIP from Session 4 onwards (*p <* .024, *d >* 0.45). Further, RTs in the practice sequence were significantly shorter in the practice hand than in the transfer hand from Session 4 onwards in AEP (*p <* .001, *d >* 0.7) and AIP (*p <* .044, *d >* 0.3). These results indicate effector-dependent representations in both AEP and AIP. Additionally, effector-dependent representations were acquired earlier and stronger in AEP than in AIP.

## 5 Discussion

The aim of the present study was to investigate the types of representation acquired in AIP and AEP when performed with either a kinesthetic focus or a visual focus. In both AIP and AEP, RTs indicated a general decrease in RTs in the course of learning, indicating sequence-unspecific learning. As expected, shorter RTs were observed in the practice sequence than in the different sequence indicating sequence-specific learning which was observed in both hands. Such effector-independent visual-spatial sequence representations were acquired in AEP and AIP. Further, RTs were shorter in the practice sequence in the practice hand than in the transfer hand indicating effector-dependent representations in AEP and AIP. In the transfer hand in Session 12, shorter RTs in the mirror sequence than in the different sequence indicated effector-independent intrinsic representations in AEP and AIP, after a break of one month. While RTs decreased, error rates increased in the course of learning particularly in the AEP groups (see supplemental material). However, error rates did not increase in the practice sequence in the practice hand. Contrary to our expectations, performance (RTs and error rates) in the tests did not significantly differ between focus groups.

### 5.1 Sequence-unspecific learning

RTs became shorter in the course of learning in all sequences of both hands of all groups indicating sequence-unspecific learning effects. This was expected, as such general learning effects have been observed in previous studies using sequential keypresses (Dahm, Weigelt, & Rieger, 2023; Wohldmann et al., 2008) showing adaptations to the task requirements. Sequence-unspecific learning may result from repeated testing, i.e., better performance in a task after testing than without previous testing of the task (Roediger & Karpicke, 2006) or stimulus-response coupling, i.e., the intensification of associations between stimuli and corresponding responses (Schneider & Shiffrin, 1977). Sequence-unspecific learning during implicit sequence learning has been shown to automatize in both, AEP and AIP (Dahm, Hyna, & Krause, 2023).

Unexpectedly, RTs in the different sequence were shorter in AEP than in AIP in the practice hand, but not in the transfer hand. In line with this, RTs in the different sequence did not significantly differ between the practice hand and the transfer hand in AIP, whereas RTs in the AEP group were shorter in the practice hand than in the transfer hand. We therefore conclude that sequence-unspecific learning was effector-dependent in AEP, but not in AIP.

### 5.2 Types of representation

In both hands, RTs were shorter in the practice sequence than in the different sequence indicating *effector-independent visual-spatial representations* after AEP and AIP. This was expected, because effector-independent visual-spatial representations have been observed in AIP in various instances (Dahm & Rieger, 2023; Dahm, Weigelt, & Rieger, 2023; Ingram, Kraeutner, Solomon, Westwood, & Boe, 2016; Wohldmann et al., 2008), indicating that flexible representations can be acquired in both, AEP and AIP.

In the practice sequence, shorter RTs in the practice hand than in the transfer hand indicated *effector-dependent representations* in both AEP and AIP. One may argue that shorter RTs in the practice hand than in the transfer hand do not necessarily indicate effector-dependent sequence representations, but may stem from sequence-unspecific learning in the hand used during practice. However, this effect was only observed for the practice sequence, but not for the different sequence. Hence, this sequence-specific finding cannot be attributed to sequence-unspecific learning in the practice hand. Effector-dependent representations in AIP have previously been observed using a serial reaction time task (Dahm & Rieger, 2023), but not in deterministic sequence learning tasks, in which the sequence is available to participants and always starts at the same starting point (Dahm, Weigelt, & Rieger, 2023; Land et al., 2016). Is was argued that effector-dependent representations are not (or to a smaller degree) acquired in AIP because actual kinesthetic feedback is not available in AIP (Dahm, Weigelt, & Rieger, 2023; Ingram et al., 2019). The present data indicate that despite of the absence of actual feedback, effector-dependent representations were acquired in AIP. We speculate that this was particularly triggered by focusing on kinesthetic aspects of the task during imagery which was observed in both AIP groups (despite the focus instruction). Alternatively, the enhanced stimulus-response coupling observed in the SRT (Dahm, Hyna, & Krause, 2023) may be effector-dependent.

By using different stimuli for both the mirror and practice sequence in the transfer hand, we aimed to optimize the opportunities to find *effector-independent intrinsic representations* that are used when performing mirror sequences requiring homologous muscles of the transfer hand. Indeed, in the transfer hand in the follow-up test, RTs were shorter in the mirror sequence than in the different sequence. This was significant in AEP and in AIP. Hence, effector-independent intrinsic representations of the sequence appeared only after a one-month break without practice. Usually, effector-independent intrinsic representations are expected to evolve at late stages of practice (Panzer et al., 2009). The present results however reveal that consolidation processes (Debarnot et al., 2019; Meier & Cock, 2014) are needed to develop effector-independent intrinsic representations in both AEP and AIP.

Because RTs may be prone to a potential speed-accuracy trade-off, error rates were analyzed in addition to RTs (see supplemental material). Indeed, error rates increased in the course of learning which stands in contrast to the learning effects observed in RTs. However, error rates did not increase in all conditions and groups. Strikingly, error rates increased in AEP, but not significantly in AIP. Hence, AEP reinforced participants’ intention to perform as fast as possible by disregarding potential errors in the course of learning. Further, in AEP error rates did not increase in the course of learning in the practice sequence in the practice hand. This goes in line with the faster RTs in this condition. Possibly, after AEP the acquired sequence representations interfered with the execution of other sequences, whereas after AIP the acquired representations remained more flexible (Wohldmann et al., 2008).

### 5.3 Do modality instructions matter?

Participants subjective ratings indicated that modality instructions influenced the content of imagination as participants focused more strongly on the instructed modality relative to the other modality that was not instructed. However, in execution the modality instruction did not significantly influence the reported modality focus. It may be more difficult to adhere to modality instructions during execution, because tasks characteristics may evoke attention to certain aspects to different degrees. At least, it may be difficult to adhere to focus instructions in the long run. Note that we asked participants to report their modality focus only in Session 10, and not earlier during the experiment as we did not want to influence subsequent AIP by emphasizing other modalities in the report questions. In imagination, it might be easier to flexibly adjust the modality focus by paying attention to different aspects of the task. This flexibility may however not come without costs: RTs were generally slower when focusing on visual aspects than when focusing on kinesthetic aspects in AIP, but not in AEP (see the analysis of RTs during practice in the supplement). Thus, even though it is easier to focus on aspects of a task which may not be the “natural” ones to focus on in AIP than in AEP, this requires more resources than to focus on aspects of a task which are evoked by it.

Apart from the effects of modality on RTs during practice, the acquisition of sequence representations (performance in the tests) did not significantly differ depending on focus instructions as we had expected. Similar performance improvements after kinesthetic and visual imagery have also been observed in a ball-throwing task (Taktek, Zinsser, & St-John, 2008). It should be noted that the focus groups reported a stronger focus on the required modality than in the other modality condition in AIP, but still both modalities were used in all groups. This provides further evidence that action-imagery is multi-modal (Dahm, 2020; Krüger et al., 2022; Lacey & Lawson, 2013) and that focusing on one modality more strongly does not exclude other modalities from imagination. Hence, independent from the instructions, relevant aspects and modalities of a task are always integrated in imagination. Such a simultaneous focus on both, kinesthetic and visual aspects of the action may then evolve in performance improvements (Taktek et al., 2008). Alternatively, differences may only arise taking into account individual preferences (kinesthetic or visual) of the participants (Guillot, Collet, & Dittmar, 2004) which were not considered in the group assignment of the present study.

### 5.4 Limitations and perspectives

The question whether participants comply with instructions to imagine an action always arises in investigations of action imagery, as imagery itself cannot be directly observed (Dahm, 2020). In the present study, all participants, including those in the imagery groups, pressed the shift key during practice, allowing us to measure the duration of their imagery. Similar to previous studies (Dahm & Rieger, 2023; Dahm, Weigelt, & Rieger, 2023), the durations during practice showed an improvement in performance over time in both the action imagery practice (AIP) and action execution practice (AEP) groups. This suggests that participants likely followed the imagery instructions during practice. Moreover, the sequence-specific learning effects observed after AIP support the assumption that most participants adhered to the imagery instructions during practice.

One may argue, that some participants would naturally prefer the third-person perspective over the first-person perspective (Callow & Roberts, 2012; Liu, Lai, Fong, & Bissett, 2019; Spittle & Morris, 2007). Hence, it may have been difficult for them to perform imagery from a first-person perspective as instructed. However, we think difficulties with the first-person perspective are rather unlikely in the present task, as any third person perspective would have the result that some essential element of the apparatus is not fully visible or reversed, which would interfere with task performance. For instance, from the ‘gaming perspective’ behind oneself with some distance, one may see the stimuli on screen but also one’s own back (instead of the keyboard and the hand movements).

It is worth noting that the pressing of the space key during practice may be considered a confounding factor that could have hindered learning due to the costs associated with dual-tasking (Röttger, Zhao, Gaschler, & Haider, 2021). However, since this was consistent across all practice groups, it does not pose a confound in itself. Nonetheless, practice in the AIP groups, which involved thumb movements, may be seen as a combination of AIP and AEP. Despite this, the observed sequence-specific learning effects in AIP cannot be attributed to the additional presses of the shift key.

Using the intermanual transfer paradigm does not allow to distinguish whether effector-dependent representations are based on visual stimulus learning, visual learning of the response keys, or motor learning associated with the fingers. To investigate whether the observed learning effects in AIP and AEP differ regarding visual-spatial representations of the stimuli, visual-spatial representations of the response keys, or motor representations of the fingers, future studies may use a crossed-hands transfer paradigm (Bird & Heyes, 2005; Willingham et al., 2000).

## 6 Conclusion

In a serial reaction time task, focusing on one particular modality does affect the imagery process itself but does not affect sub-sequent performance improvements and the type of representation acquired during AIP. General sequence-unspecific learning was observed in all sequences and hands. Sequence-specific representations evolved in AEP and AIP. Sequence-specific representations were predominantly effector-independent visual-spatial in nature. However, effector-independent intrinsic representations may also develop in both, AIP and AEP, as indicated by the follow-up test. Interestingly, sequence-specific effector-dependent representations were observed in both practice types, but earlier and stronger in AEP than in AIP. The observed evidence for sequence-specific effector-dependent representations in AIP is particularly interesting, because it shows that even motorically stored information such as effector-dependent representations can be acquired using AIP. Most likely, performance improvements in AIP were caused by the use of internal models that predict the action consequences (Rieger et al., 2023).

## Supplementary Material

Supplemental Material

## Figures and Tables

**Table 1:** Sociodemographic data of the experimental groups. To compare the practice groups, a X^2^ Test was calculated for the distribution of sex and ANOVAs with the factor group (kinesthetic action-imagery practice, visual action-imagery practice, kinesthetic action-execution practice, and visual action-execution practice) were computed for the remaining variables.

	Action-imagery practice		Action-execution practice		*p*
	Kinesthetic	Visual	Kinesthetic	Visual	
Sex, *N*_female_ / *N*_male_	17 / 9	14 / 9	17 / 8	17 / 11	.42
Age, *M* ± *SD*	24.8 ± 5	24.3 ± 4.3	24.1 ± 4.1	23.7 ± 3.6	.814
Laterality index, *M* ± *SD*	94 ± 11	92 ± 12	96 ± 10	95 ± 11	.644
External visual imagery, *M* ± *SD*	1.6 ± 0.6	1.8 ± 0.6	1.9 ± 0.8	2 ± 0.7	.173
Internal visual imagery, *M* ± *SD*	1.5 ± 0.5	1.6 ± 0.5	1.7 ± 0.6	1.8 ± 0.6	.144
Kinesthetic imagery, *M* ± *SD*	1.7 ± 0.6	1.6 ± 0.6	1.8 ± 0.9	1.7 ± 0.5	.817

**Table 2:** Overview of the experimental sessions.

Session	1	2	3	4	5	6	7	8	9	10	11	12
Test	✓	✓	⨯	✓	⨯	⨯	✓	⨯	⨯	⨯	✓	✓
Practice	✓	✓	✓	✓	✓	✓	✓	✓	✓	✓	⨯	⨯

**Table 3:** Statistical values of the ANOVA on reaction times. The ANOVA was conducted with the factors practice (action-imagery, action-execution), focus (kinesthetic, visual), hand (practice, transfer), sequence (practice, mirror, different), and test (1, 2, 3, 4, 5, 6).

	*F*	*df1, df2*	*p*	${\text{η}}_{p}^{2}$
Practice	3.7	1, 98	.056	0.04
Focus	0.5	1, 98	.473	0.01
Hand	4.8	1, 98	.031	0.05
**Sequence**	**121.3**	**1.8, 177**	**<.001**	**0.55**
**Test**	**199.9**	**3.6, 355.8**	**<.001**	**0.67**
Practice x Focus	0.2	1, 98	.634	<0.01
**Practice x Hand**	**11.1**	**1, 98**	**.001**	**0.1**
**Practice x Sequence**	**8.1**	**1.8, 177**	**.001**	**0.08**
Practice x Test	1.5	3.6, 355.8	.209	0.02
Focus x Hand	0.5	1, 98	.479	0.01
Focus x Sequence	1.9	1.8, 177	.153	0.02
Focus x Test	2	3.6, 356	.103	0.02
**Hand x Sequence**	**94.8**	**1.6, 159.1**	**<.001**	**0.49**
**Hand x Test**	**3.9**	**4.9, 482.3**	**.002**	**0.04**
**Sequence x Test**	**50.6**	**7.9, 774.8**	**<.001**	**0.34**
Practice x Focus x Hand	0.6	1, 98	.44	0.01
Practice x Focus x Sequence	1.8	1.8, 177.4	.178	0.02
Practice x Focus x Test	0.5	3.6, 355.8	.696	0.01
**Practice x Hand x Sequence**	**5.7**	**1.6, 159.1**	**.007**	**0.06**
**Practice x Hand x Test**	**4**	**4.9, 482.3**	**.001**	**0.04**
**Practice x Sequence x Test**	**4**	**7.9, 774.8**	**<.001**	**0.04**
Focus x Hand x Sequence	1.4	1.6, 159.1	.244	0.01
Focus x Hand x Test	1.6	4.9, 482.3	.155	0.02
Focus x Sequence x Test	1.1	7.9, 774.8	.345	0.01
**Hand x Sequence x Test**	**22.1**	**7.7, 752.8**	**<.001**	**0.18**
Practice x Focus x Hand x Sequence	0.9	1.6, 159	.398	0.01
Practice x Focus x Hand x Test	1.3	4.9, 482.3	.245	0.01
Practice x Focus x Sequence x Test	0.5	7.9, 774.8	.873	0.01
**Practice x Hand x Sequence x Test**	**2.2**	**7.7, 752.8**	**.03**	**0.02**
Focus x Hand x Sequence x Test	1.3	7.7, 752.8	.525	0.01
Practice x Focus x Hand x Sequence x Test	1.4	7.7, 752.8	.187	0.01

*Note.* Significant effects are in bold.

## Data Availability

The author confirms that the data supporting the findings of this study are available within the article and its supplementary material: https://osf.io/puzr3/. Determination of the sample size, all data exclusions, all manipulations, and all measures in the study are reported in the manuscript and its supplemental material. The R packages ‘tidyverse’ (Wickham et al., 2019) and ‘ggplot2’ (Wickham, 2016) have been used for data restructuring and figure generation.

## Footnotes

1 In previous studies, modality and perspective have sometimes been confounded (Hall & Martin, 1997; Isaac, Marks, & Russell, 1986). Visual imagery can be performed from a first-person perspective (through your own eyes) and from a third-person perspective (from another external viewpoint). Such a distinction between perspectives is not possible for kinesthetic imagery (Dahm, 2020; Roberts et al., 2008; Williams et al., 2012). To avoid confounding modality and perspective, visual imagery was performed from a first-person perspective in the present study.
