# Supplementary material for: Kinesthetic vs. visual focus: No evidence for effects of practice modality in representation types after action imagery practice and action execution practice
Source: Hum Mov Sci. Author manuscript; Available in PMC 2023 Dec 13. (PMC7615372; doi:10.1016/j.humov.2023.103154)
Supplement: Supplemental Material [file EMS192560-supplement-Supplemental_Material.pdf]

SUPPLEMENTAL MATERIAL

Kinesthetic vs. visual focus: No evidence for effects of practice modality in representation types after action imagery practice and action execution practice

Stephan F. Dahm [1] & Martina Rieger [2]
[1] Universität Innsbruck, Department of Psychology, Innsbruck, Austria
[2] UMIT TIROL – private university of health sciences and health technology, Institute of Psychology, Hall in Tyrol, Austria

Corresponding author
Stephan Frederic Dahm
Universität Innsbruck, Department of Psychology
Innrain 52, 6020 Innsbruck, Austria
Email: stephan.dahm@uibk.ac.at

Overview

This document includes additional analyses that may be of interest to some readers. In particular, we report secondary dependent variables that were collected, but are not reported in the final version of the manuscript. First, we provide a detailed description of participant exclusions. Second, we report an analysis of the error rates that is analogous to the RTs in the manuscript. Third, we report reaction times (RTs) that were collected during action execution practice (AEP) and action imagery practice (AIP). Fourth, we report associations between stimuli that could have influenced the perception of the stimuli in the transfer hand. Fifth, we report participants sequence knowledge as assessed with free generation and recognition tests.

1. **Participant exclusions**

Due to technical issues, the data sets of 8 participants were incomplete and therefore not analyzed. Of 118 complete data sets, sixteen were excluded from analysis. Four participants misunderstood the mapping of the visual stimuli and response locations. Three participants had unusually long RTs during practice (> 3 *SD* above the group mean). Nine participants had more than one block with error rates above 30%. Of those, six participants had consecutive errors in combination with very short RTs (below 200 ms) which indicated that participants just pressed keys random keys instead of responding to the stimuli. The other three participants with high errors rates had very long RTs (over 1500 ms) on errors indicating distraction.

2. **Error rates**

Boxplots of error rates are shown in Figure SM1. A mixed-model ANOVA with the between factors practice (AIP, AEP) and focus (kinesthetic, visual) and the within factors hand (practice, transfer), sequence (practice, mirror, different), and test (Sessions: 1, 2, 4, 7, 11, 12) was conducted on error rates. The results of the ANOVA can be seen in Table SM1.

Sequence-unspecific general learning effects and control comparisons

Apart from the interaction between focus, hand, and test, neither the main effect of *focus* nor any interactions with it became significant. The interaction revealed significant differences between the focus groups. The error rates were significantly higher in the kinesthetic practice groups than in the visual practice groups in the practice hand in Session 2 (p = .006, d = 0.32) and Session 3 (p = .021, d = 0.26) and in the transfer hand in Session 1 (p = .016, d = 0.28), Session 2 (p = .008, d = 0.31), and Session 11 (p = .002, d = 0.36). Hence, there were some slight differences between groups, that were however not provoked by the manipulation.

Comparisons in Session 1: In AEP and AIP, error rates did not significantly differ between sequences (p > .201, d < 0.27) or between hands (p > .097, d < 0.24). Further, error rates did not significantly differ between the groups in all sequences (p = .165, d < 0.28). Hence, performance did neither differ significantly between sequences, hands, nor between practice groups before practice started.

The significant main effect *test* was modified by the significant interaction between test and practice, the significant interaction between test, practice, and hand and the significant interaction between test, sequence, and hand. Therefore, we compared the error rates between subsequent tests for each practice group, hand, and sequence.

In AEP, the error rates were higher in Session 11 than in Session 1 in all sequences and both hands (p < .001, d > 0.6), except for the practice sequence in the practice hand (p = .211, d = 0.2). Similarly, in AIP, the error rates were higher in Session 11 than in Session 1 in all sequences and both hands (p < .013, d > 0.4), except for the practice sequence in the practice hand (p = .238, d = 0.2) and the practice sequence in the transfer hand (p = .054, d = 0.3). These effects stand in contrast to the sequence-unspecific learning effects in RTs and thus indicate a potential speed-accuracy tradeoff in general task performance.

Sequence-specific learning effectsThe significant main effect of *sequence* was modified by the significant interactions between sequence and test, the significant interaction between sequence, practice, and hand, and the significant interaction between sequence, test, and hand. Therefore, we compared the error rates between sequences for each practice group, hand, and test. As in the analysis of RTs, we visualized the sequence-specific learning effects in error rates with the sequence-learning index (different sequence - sequence of interest) (Dahm & Rieger, 2023; Kraeutner et al., 2016) in Figure SM2.

After practice (in the first 10 sessions), we found the following in Session 11: In the practice hand, significantly lower error rates in the practice sequence than in the different sequence indicated sequence-specific learning in AEP ($p < .001$, $d = 0.8$), but not in AIP ($p = .134$, $d = 0.3$). In the transfer hand, error rates did not significantly differ between the practice sequence and the different sequence in AEP ($p > .999$, $d = 0.1$) and AIP ($p > .999$, $d = 0.1$). Hence, the error rates showed no evidence for effector-independent representations.

Further, comparisons of the practice sequence between hands resulted in significantly lower error rates in the practice hand than in the transfer hand in AEP ($p = .002$, $d = 0.5$), but not in AIP ($p = .787$, $d = 0.04$). In contrast, in the different sequence the error rates were significantly higher in the practice hand than in the transfer hand ($p = .027$, $d = -0.3$). Hence, the error rates showed evidence for effector-dependent representations in AEP, but not in AIP.

Comparisons between practice groups in Session 11 showed that error rates were significantly higher in AEP than in AIP in the different sequence in the practice hand ($p = .005$, $d = 0.6$) and in the transfer hand ($p = .028$, $d = 0.4$). This was not significant in the practice sequence in the practice hand ($p = .805$, $d = 0.05$). Hence, in the unpracticed conditions, the error rates increased in the course of learning in AEP, but not in AIP.

One-month follow-up test in Session 12: The effector-dependent representations AEP were replicated by significantly lower error rates in the practice sequence in the practice hand than in the transfer hand ($p = .019$, $d = 0.3$). Again, the error rates in the different sequence were reverse, i.e., were significantly higher in the practice hand than in the transfer hand ($p = .035$, $d = -0.3$). This indicates that effector-dependent representations were maintained after a one-month interval without practice.

Figure SM1. Boxplots of error rates (in %) depending on hand (practice, transfer), sequence (practice, mirror, different), and test (1, 2, 4, 7, 11, 12) separately for the groups which differed in practice (action-execution practice in dark, action-imagery practice in light) and focus (kinesthetic in red, visual in blue).

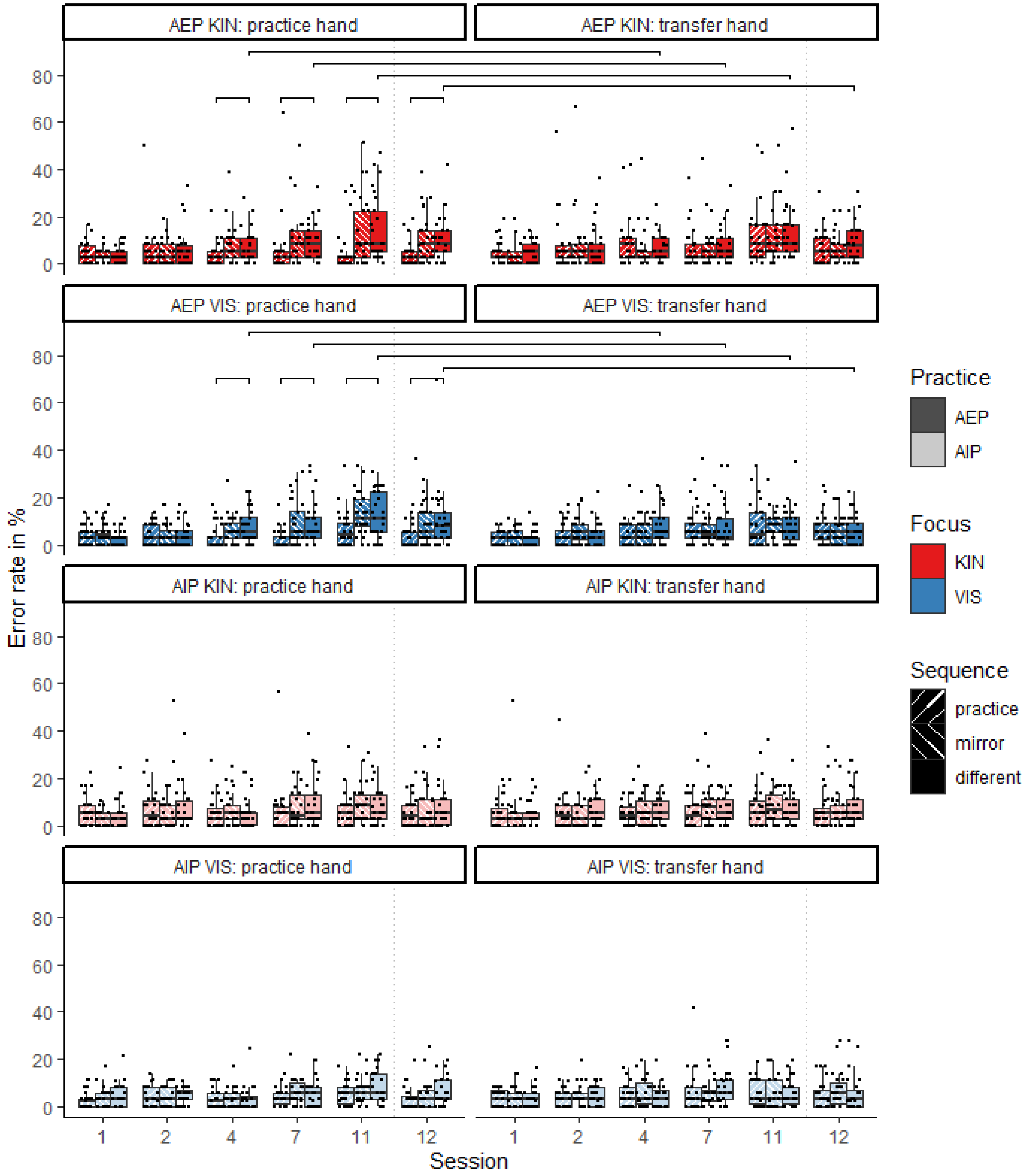

Figure SM2. Means of the sequence learning indexes (different sequence – sequence of interest) for percentage of errors depending on hand (practice, transfer), sequence (practice, mirror), and test (1, 2, 3, 4, 5, 6) separately for the groups which differed in practice (action-execution in dark, action-imagery in light) and focus (kinesthetic in red, visual in blue).

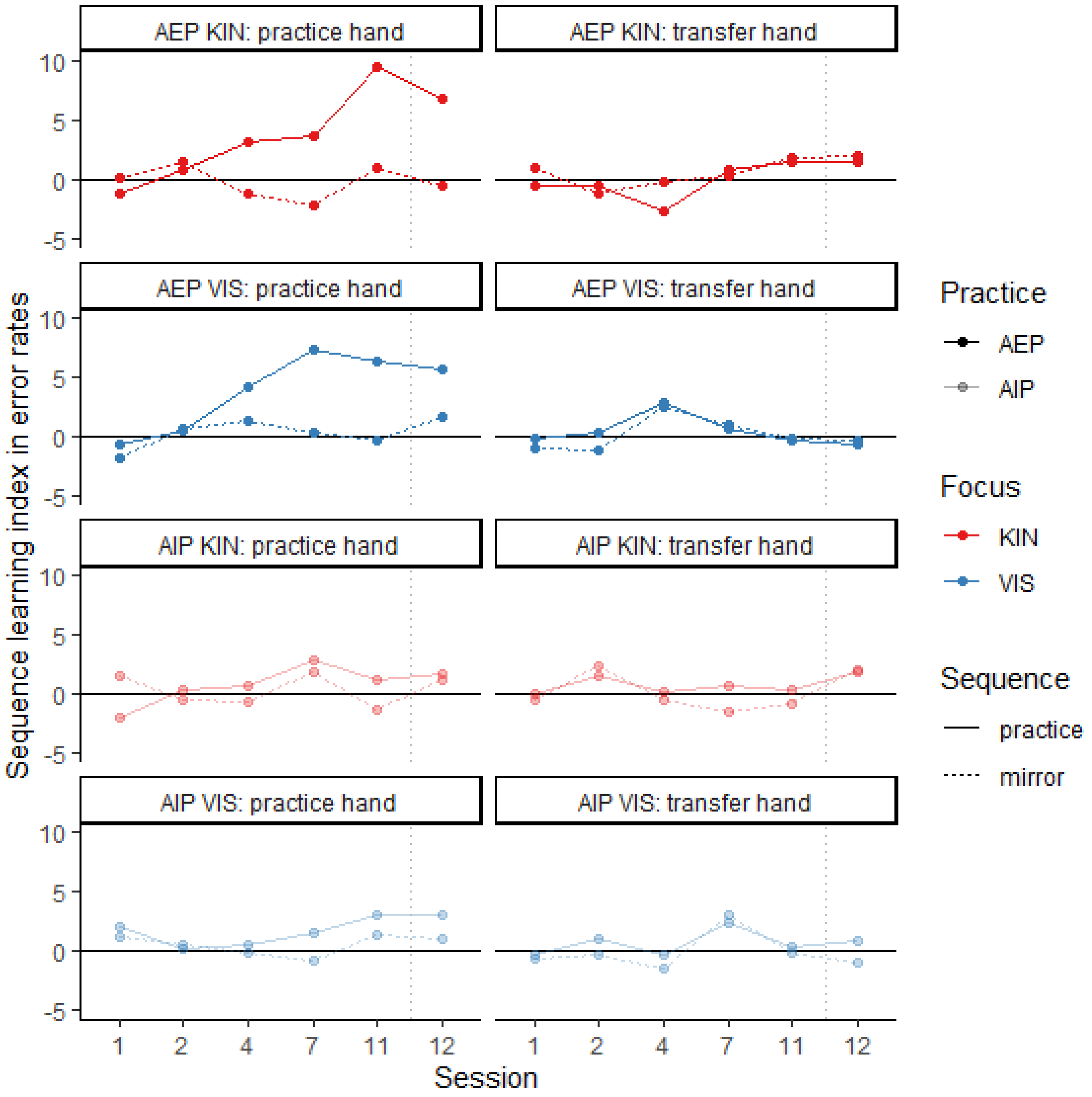

Table SM1

Statistical values of the ANOVA on error rates. The ANOVA was conducted with the factors practice (action-imagery, action-execution), focus (kinesthetic, visual), hand (practice, transfer), sequence (practice, mirror, different), and test (1, 2, 3, 4, 5, 6).

	F	*df1, df2*	*p*	η^2_p
Practice	1.7	1, 98	.19	.02
Focus	3.2	1, 98	.077	.03
Hand	0.2	1, 98	.689	<.01
**Sequence**	**21.6**	**2, 196**	**<.001**	**.18**
**Test**	**18.9**	**4.1, 404.9**	**<.001**	**.16**
Practice x Focus	<0.1	1, 98	.991	<.01
Practice x Hand	0.3	1, 98	.567	<.01
Practice x Sequence	3.1	2, 196	.05	.03
Practice x Test	3.1	4.1, 404.9	.014	.03
Focus x Hand	0.3	1, 98	.602	<.01
Focus x Sequence	0.3	2, 196	.752	<.01
Focus x Test	0.5	4.1, 404.9	.724	.01
**Hand x Sequence**	**14.4**	**2, 196**	**<.001**	**.13**
Hand x Test	1.3	4.7, 459	.28	.01
**Sequence x Test**	**3.6**	**9.3, 908.6**	**<.001**	**.04**
Practice x Focus x Hand	2.6	1, 98	.111	.03
Practice x Focus x Sequence	<0.1	2, 196	.973	<.01
Practice x Focus x Test	0.2	4.1, 404.9	.956	<.01
**Practice x Hand x Sequence**	**10.1**	**2, 196**	**<.001**	**.09**
**Practice x Hand x Test**	**2.5**	**4.7, 459**	**.033**	**.03**
Practice x Sequence x Test	1.1	9.3, 908.6	.374	.01
Focus x Hand x Sequence	0.3	2, 196	.714	<.01
**Focus x Hand x Test**	**2.4**	**4.7, 459**	**.043**	**.02**
Focus x Sequence x Test	0.9	9.3, 908.6	.515	.01
**Hand x Sequence x Test**	**3.3**	**9.4, 918.3**	**.001**	**.03**
Practice x Focus x Hand x Sequence	0.6	2, 196	.548	.01
Practice x Focus x Hand x Test	0.3	4.7, 459	.907	<.01
Practice x Focus x Sequence x Test	1	9.3, 908.6	.404	.01
Practice x Hand x Sequence x Test	1.6	9.4, 918.3	.109	.02
Focus x Hand x Sequence x Test	1.1	9.4, 918.3	.374	.01
Practice x Focus x Hand x Sequence x Test	1	9.4, 918.3	.417	.01

Note. Significant effects are in bold.

3. Reaction times during practice

To analyze RTs during practice, median RTs of each session were calculated. RTs during practice were calculated using the responses on the additional shift key in both AIP and AEP. Boxplots of RTs during practice are shown in Figure SM3. A mixed-model ANOVA with the between-factors practice (AIP, AEP) and focus (kinesthetic, visual) and the within-factor session (1, 2, 3, 4, 5, 6, 7, 8, 9, 10) was conducted on RTs during practice.

The significant main effect practice, F (1, 98) = 14.4, p < .001, η_p^2 = .13, was modified by the significant interaction between practice and focus, F (1, 98) = 6.1, p = .015, η_p^2 = .06. RTs were significantly longer in AIP than in AEP with visual focus (p < .001), but with kinesthetic focus (p = .349). The significant main effect session, F (2.6, 258.8) = 139.4, p < .001, η_p^2 = .59, indicated significant improvements between successive sessions (p_{min} = .044), except between Session 7 and 8 (p >.99) and between Session 9 and 10 (p >.99). The significant interaction between session and focus, F (2.6, 258.8) = 3.8, p = .015, η_p^2 = .04, indicated that the differences between sessions were significantly larger in the visual focus groups (ΔM = 29 ms) than in the kinesthetic focus groups (ΔM = 21 ms, p = .022). The remaining effects were not significant, focus: F (1, 98) = 1.2, p = .28, η_p^2 = .01; practice x session: F (2.6, 258.8) = 2.3, p = .089, η_p^2 = .02; practice x focus x session: F < 1.

Figure SM3. Boxplots of reaction times during practice (in ms) depending on session (1, 2, 3, 4, 5, 6, 7, 8, 9, 10) separately for the groups which differed in practice (AIP: action-imagery practice, AEP: action-execution practice) and focus (kinesthetic, visual).

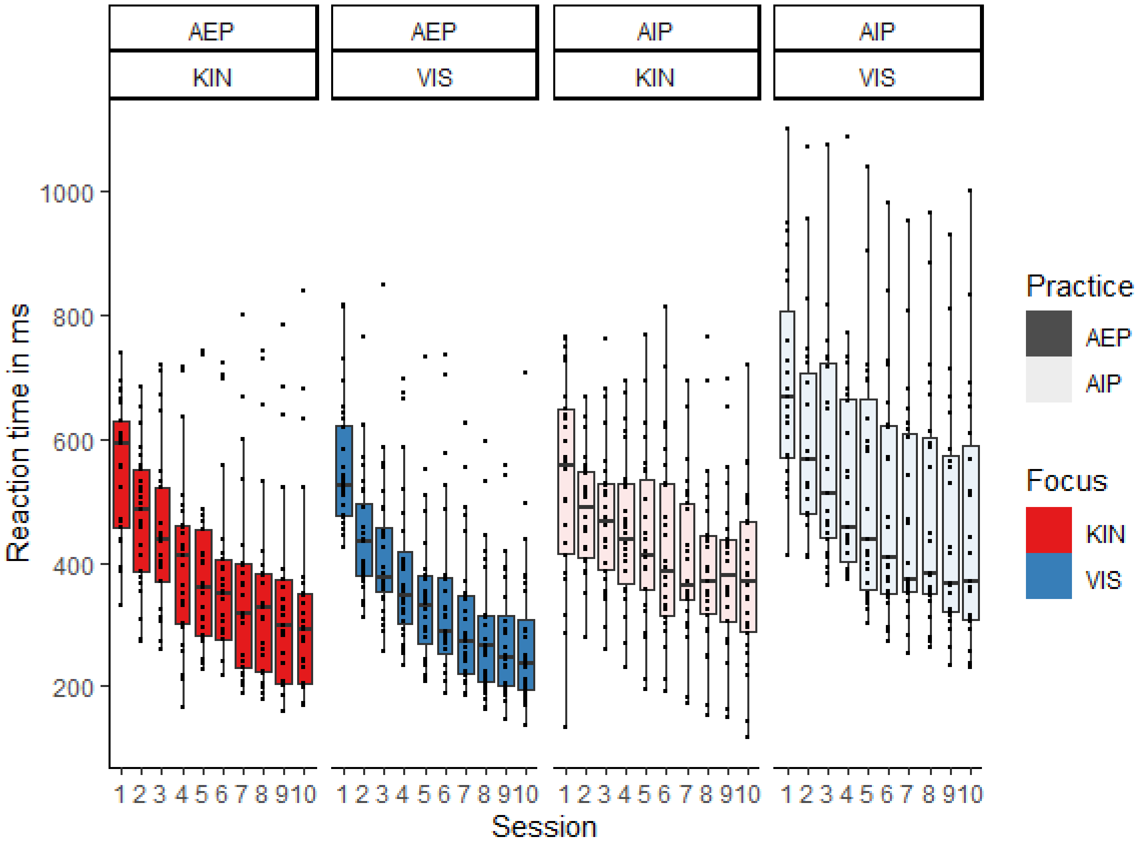

RTs during practice revealed differences between the focus groups in both AIP and AEP. In line with previous studies, RTs were generally longer in imagination than in execution (Dahm & Rieger, 2016b, 2016a), probably due to a higher cognitive load in imagery than in execution (Glover & Baran, 2017). More importantly for the present work, the focus on modalities influenced the timing during practice. In both, AEP and AIP with a kinesthetic focus, in the course of the experiment RTs decreased less than with a visual focus. This goes in line with results showing that compared to an external focus (on targets or objects in the environment), an internal focus of attention (on one's body) hampers performance improvements (Chua et al., 2021). Alternatively, it could be argued that in the first practice session the visual focus groups performed slower than the kinesthetic focus groups which left more space for improvement in the visual focus groups.

4. Preferred association between stimuli

To investigate the way in which the stimuli of the transfer hand were perceived similar to the stimuli of the practice hand, participants were asked to indicate in which one out of two stimulus pairs the stimuli were more similar to each other. If the practice and focus manipulations lead to differences in the acquired representations, this may also affect how participants perceive the stimulus material. This assessment further served to rule out stimulus learning (Koch & Hoffmann, 2000). For this, stimulus pairs were created that were either intrinsically congruent, extrinsically congruent, or incongruent. In the task, two stimulus pairs were presented at the same time on the left and right side of the screen (see Figure SM4). Participants were asked to decide which of the two stimulus pairs they perceived as more similar by clicking with the mouse on the respective item pair. This included twelve trials, in which each type of stimulus pair was paired with each of the other types of stimulus pair four times.

Figure SM4. Exemplary depiction of two trials of assessment of preferred associations between stimuli. In the upper part of the figure an intrinsically congruent stimulus pair is compared to an extrinsically congruent stimulus pair. In the lower part of the figure an extrinsically congruent stimulus pair is compared to an incongruent stimulus pair. Note that the description (in italics) was not shown to participants.

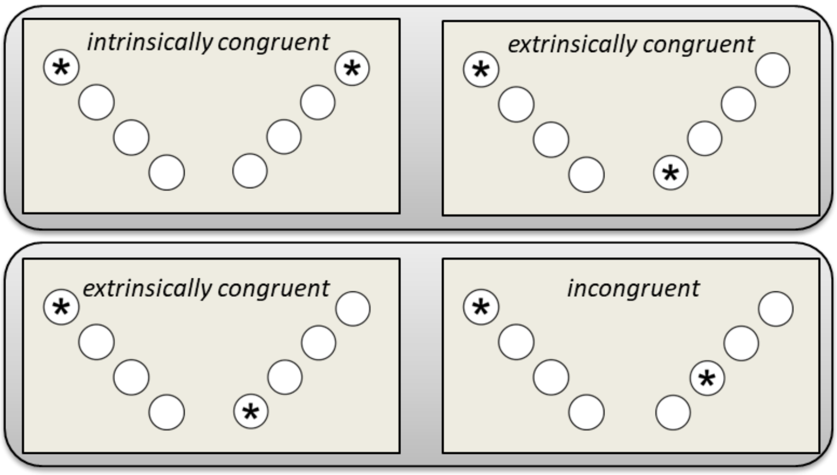

Associations between stimuli were analyzed using the number of chosen extrinsically congruent, intrinsically congruent, and incongruent stimulus pairs as a dependent variable. Boxplots of the number of selected stimulus pairs are shown in Figure SM5. A mixed-model ANOVA was calculated with the between factors practice (AIP, AEP) and focus (kinesthetic, visual) and the within factor stimulus pair (extrinsic congruent, intrinsic congruent, incongruent). The significant main effect of stimulus pair, F (2, 196) = 79.6, p < .001, η_p^2 = .45, indicated that intrinsic congruent stimulus pairs (M = 6) were more often selected than extrinsic congruent stimulus pairs (M = 4.2, p < .001), which in turn were more often selected than incongruent stimulus pairs (M = 1.8, p < .001). All remaining interactions with the factor stimulus pair were not significant, F < 1.

Figure SM5. Boxplots of the number of selected extrinsic congruent, intrinsic congruent and incongruent stimulus pairs, separately for the groups which differed in practice (AIP: action-imagery practice, AEP: action-execution practice) and focus (kinesthetic, visual).

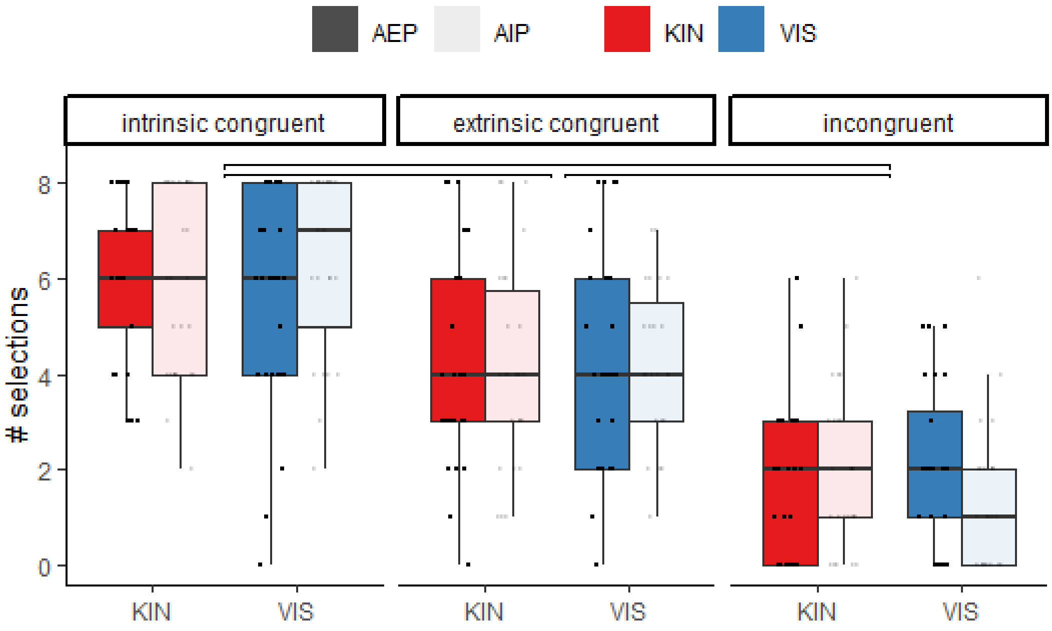

The analysis of the *preferred associations* between stimulus pairs showed that participants of all groups had a preference for intrinsic congruent stimulus pairs over extrinsic congruent stimulus pairs. Intrinsic congruent matches coincided with mirror images (and mirror sequences) in the practice and transfer hand, whereas extrinsic congruent matches coincided with visual-spatial (left/right) images (and sequences) in the practice and transfer hand (Figure 2). However, this may have been independent from practice in the present task, as people prefer symmetry over asymmetry in many domains, e.g., the perception of shapes (Bertamini et al., 2019) and the perception of bimanual movements (Mechsner et al., 2001). It is unlikely that these preferences for intrinsic congruent stimulus pairs (rather than extrinsic congruent stimulus pairs) resulted from practicing the present task because the preference for stimulus pairs did not coincide with the pattern of results observed in RTs. However, the preference for

intrinsically congruent stimuli may have contributed to the development of effector-independent intrinsic representation after some time of consolidation, for which we observed some evidence in the follow-up tests.

In any case, the preferred associations between stimuli for the left and right hand (intrinsically congruent) differed from the predominant representation of the sequence (visual-spatial, extrinsically congruent). Hence, the effector-independent visual-spatial representations acquired in AEP and AIP were most likely based on anticipation of response locations rather than on anticipation of stimuli (Koch & Hoffmann, 2000). Most likely, participants did not focus solely on kinesthetic aspects of the task, but also on tactile sensations when pressing the keys. The latter may have promoted anticipations of the response locations.

5. Free generation and Recognition tests

In addition, we investigated whether participants become aware of the sequence. Although the task did not involve explicit instructions about a sequence, participants' may become aware of the sequence in the course of learning (Kraeutner et al., 2016, 2017). Because we used random starting points in a 12-element sequence, we did not expect participants to be able to freely generate the practice sequence (Dahm et al., 2023). However, recognition of the practice sequence during sequence execution may arise, as this may not only rely on explicit knowledge but also on an unspecific intuition of familiarity (Dahm et al., 2023). Most importantly, we did not expect free generation or recognition performance to differ after AEP and AIP (Dahm et al., 2023).

To investigate whether participants became aware of the practiced sequence, participants performed a *free generation* test and a *recognition test* in Session 11. In the *free generation test*, participants were told that the keypresses in the practice blocks followed a particular sequence. They were asked to remember and execute the practice sequence without visual stimuli. This was followed by a *recognition* test in which the practice sequence, the visual-spatial copy of the practice sequence, and both different sequences were performed once. After each sequence, participants rated whether the performed sequence coincided with the practice sequence (1 – "very unlikely" to 9 – "very likely"). The order of the four sequences in the recognition test was randomized.

To analyze free generation performance, we calculated the number of triplets in the free generation test that were compatible with the practice sequence and the mirror sequence (Bird & Heyes, 2005). This indicates the amount of explicit knowledge of the sequence structure (Bird & Heyes, 2005; Dahm et al., 2023). A mixed-model ANOVA was calculated with the between factors practice (AIP, AEP) and focus (kinesthetic, visual) and the within factor sequence (practice, mirror) on the number of matching triplets. The significant main effect of sequence, F (1, 98) = 24.4, p < .001, η_p^2 = .2, indicated significantly more matches with the practice sequence (M = 4.7, SE = 0.2) than with the mirror sequence (M = 3.2, SE = 0.2). All remaining effects were not significant: practice x focus x sequence: F (1, 98) = 1.7, p = .196, η_p^2 = .02; all others: F < 1.

Boxplots of recognition performance are shown in Figure SM6. To analyze recognition performance in the practice hand, a mixed-model ANOVA with the between factors practice (AIP, AEP) and focus (kinesthetic, visual) and the within factor sequence (practice, mirror, different) was calculated on the rating that a performed sequence corresponded with the practice sequence. The significant main effect of sequence, F (2, 196) = 44.1, p < .001, η_p^2 = .31, indicated significantly higher ratings for the practice sequence (M = 6.9) than for the mirror sequence (M = 4.6, p < .001) and different sequence (M = 4.9, p < .001). The latter did not significantly differ from each other (p = .457). The significant interaction between sequence and practice, F (2, 198) = 4.7, p = .01, η_p^2 = .05, indicated that these differences between practice sequence and the other sequences were significantly larger in the AEP group (M = 2.7) than in the AIP group (M = 1.5, p = .014). All remaining effects were not significant: focus: F (1, 98) = 1.9, p = .175, η_p^2 = .02; practice x focus x sequence: F (2, 196) = 1.7, p = .179, η_p^2 = .02; all others F < 1.

Figure SM6. Boxplots of the recognition ratings for the practice, mirror, and different sequence, separately for the groups which differed in practice (AIP: action-imagery practice, AEP: action-execution practice) and focus (kinesthetic, visual).

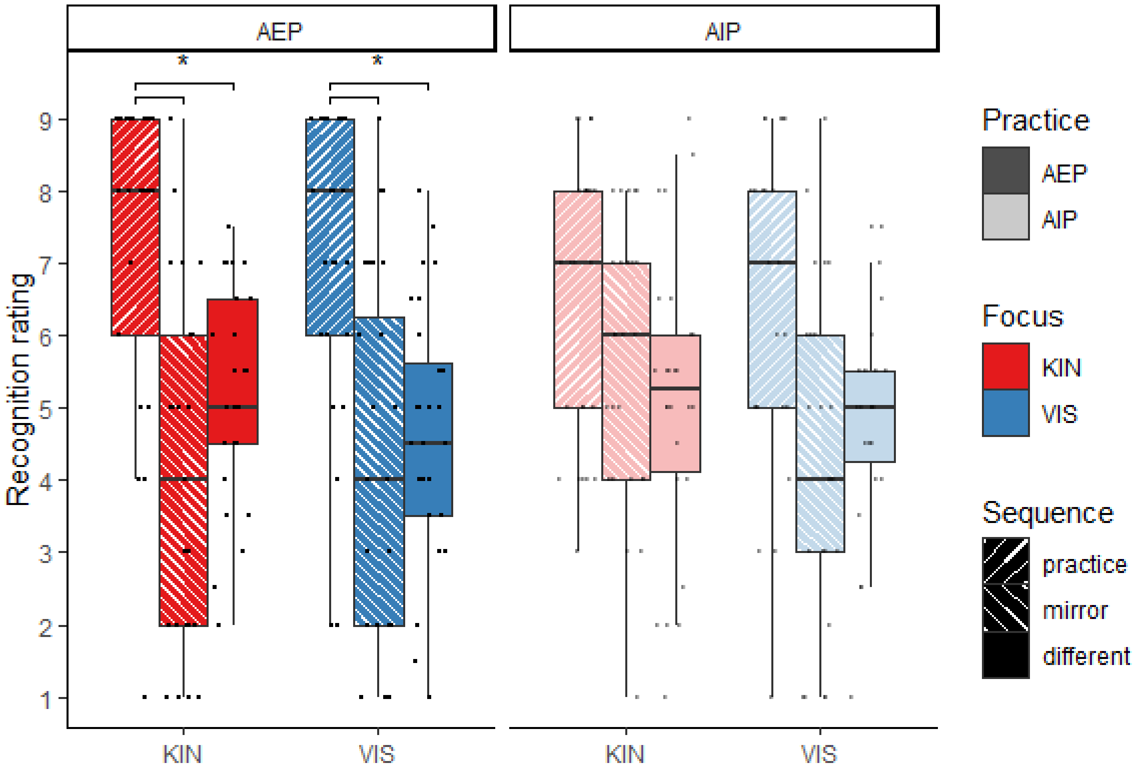

Performance in the free generation test indicated that some participants were able to reproduce parts of the twelve-element sequence. While performance in the free generation test was closer to random (in comparison to the mirror sequence as a control) than to complete reproduction (which would have been 12 triplets) in both AEP

and AIP, participants clearly recognized the practice sequence in the recognition test. Recognition of the sequence was stronger after AEP than after AIP. It has been proposed that instead of conscious sequence knowledge, participants may have an unspecific intuition in the recognition task which makes them ‘feel’ that this has been the practiced sequence (Dahm & Rieger, 2023). Such intuitive recognitions may be promoted more by AEP than by AIP.

References

Bertamini, M., Rampone, G., Makin, A. D. J., & Jessop, A. (2019). Symmetry preference in shapes, faces, flowers and landscapes. *PeerJ*, *7*, e7078. https://doi.org/10.7717/peerj.7078

Bird, G., & Heyes, C. (2005). Effector-dependent learning by observation of a finger movement sequence. *Journal of Experimental Psychology: Human Perception and Performance*, *31*(2), 262–275. https://doi.org/10.1037/0096-1523.31.2.262

Chua, L.-K., Jimenez-Diaz, J., Lewthwaite, R., Kim, T., & Wulf, G. (2021). Superiority of external attentional focus for motor performance and learning: Systematic reviews and meta-analyses. *Psychological Bulletin*, *147*(6), 618–645. https://doi.org/10.1037/bul0000335

Dahm, S. F., & Rieger, M. (2016a). Cognitive constraints on motor imagery. *Psychological Research*, *80*(2), 235–247. https://doi.org/10.1007/s00426-015-0656-y

Dahm, S. F., & Rieger, M. (2016b). Is there symmetry in motor imagery? Exploring different versions of the mental chronometry paradigm. *Attention, Perception & Psychophysics*, *78*(6), 1794–1805. https://doi.org/10.3758/s13414-016-1112-9

Dahm, S. F., & Rieger, M. (2023). Time course of learning sequence representations in action imagery practice. *Human Movement Science*, *87*, 103050. https://doi.org/10.1016/j.humov.2022.103050

Dahm, S. F., Weigelt, M., & Rieger, M. (2023). Sequence representations after action-imagery practice of one-finger movements are effector-independent. *Psychological Research*, *87*(1), 210–225. https://doi.org/10.1007/s00426-022-01645-3

Glover, S., & Baran, M. (2017). The motor-cognitive model of motor imagery: Evidence from timing errors in simulated reaching and grasping. *Journal of Experimental Psychology: Human Perception and Performance*, *43*(7), 1359–1375. https://doi.org/10.1037/xhp0000389

Koch, I., & Hoffmann, J. (2000). The role of stimulus-based and response-based spatial information in sequence learning. *Journal of Experimental Psychology: Learning, Memory, and Cognition*, *26*(4), 863–882. https://doi.org/10.1037/0278-7393.26.4.863

Kraeutner, S. N., Gaughan, T. C., Eppler, S. N., & Boe, S. G. (2017). Motor imagery-based implicit sequence learning depends on the formation of stimulus-response associations. *Acta Psychologica*, *178*, 48–55. https://doi.org/10.1016/j.actpsy.2017.05.009

Kraeutner, S. N., MacKenzie, L. A., Westwood, D. A., & Boe, S. G. (2016). Characterizing skill acquisition through motor imagery with no prior physical practice. *Journal of Experimental Psychology: Human Perception and Performance*, *42*(2), 257–265. https://doi.org/10.1037/xhp0000148

Mechsner, F., Kerzel, D., Knoblich, G., & Prinz, W. (2001). Perceptual basis of bimanual coordination. *Nature*, *414*, 69–73. https://doi.org/10.1038/35102060
